# The indole-3-carbinol cyclic tetrameric derivative CTet inhibits cell proliferation via overexpression of p21/CDKN1A in both estrogen receptor-positive and triple-negative breast cancer cell lines

**DOI:** 10.1186/bcr2855

**Published:** 2011-03-24

**Authors:** Mauro De Santi, Luca Galluzzi, Simone Lucarini, Maria Filomena Paoletti, Alessandra Fraternale, Andrea Duranti, Cinzia De Marco, Mirco Fanelli, Nadia Zaffaroni, Giorgio Brandi, Mauro Magnani

**Affiliations:** 1 Department of Biomolecular Sciences, University of Urbino 'Carlo Bo', Via Saffi 2, 61029 Urbino, Italy; 2 Department of Health and Drug Sciences, University of Urbino 'Carlo Bo', Via Saffi 2, 61029 Urbino, Italy; 3 Department of Experimental Oncology and Molecular Medicine, Fondazione IRCCS Istituto Nazionale dei Tumori, Via G. Venezian 1, 20133 Milano, Italy

## Abstract

**Introduction:**

Indole-3-carbinol (I3C), an autolysis product of glucosinolates present in cruciferous vegetables, and its dimeric derivative (3,3'-DIM) have been indicated as promising agents in preventing the development and progression of breast cancer. We have recently shown that I3C cyclic tetrameric derivative CTet formulated in γ-cyclodextrin (γ-CD) efficiently inhibited cellular proliferation in breast cancer cell lines. This study aims to analyze the mechanisms involved in the *in vitro *inhibition of cell proliferation and to evaluate the *in vivo *antitumor activity of CTet in a xenograft study.

**Methods:**

Estrogen receptor-positive MCF-7 and triple-negative MDA-MB-231 breast cancer cell lines were exposed to CTet to evaluate cell cycle perturbation (propidium iodide staining and cytofluorimetric acquisition), induction of autophagic morphological features (co-localization of LC3b autophagosome marker and LAMP2a lysosome marker by immunofluorescence) and changes in protein expression (immunoblot and microarray-based gene expression analyses). To test the *in vivo *efficacy of CTet, female athymic nude mice inoculated with MCF-7 cells were i.p. treated with 5 mg/kg/day of CTet for five days/week for two weeks and the tumor mass was externally monitored.

**Results:**

CTet induced accumulation in G2/M phase without evidence of apoptotic response induction in both cell lines tested. In triple-negative MDA-MB-231 the autophagic lysosomal activity was significantly up-regulated after exposure to 4 μM of CTet for 8 hours, while the highest CTet concentration was necessary to observe autophagic features in MCF-7 cells. The inhibition of Akt activity and p53-independent p21/CDKN1A and GADD45A overexpression were identified as the main molecular events responsible for CTet activity in MCF-7 and p53-mutant MDA-MB-231 cells. *In vivo*, CTet administration was able to significantly inhibit the growth of MCF-7 xenotransplanted into nude mice, without adverse effect on body weight or on haematological parameters.

**Conclusions:**

Our data support CTet formulated with γ-CD as a promising and injectable anticancer agent for both hormone-responsive and triple-negative breast tumors.

## Introduction

Breast cancer is one of the most common malignancies in industrialized countries and is characterized by distinct classes of tumors that respond differently to targeted therapies such as selective estrogen receptor modulator (SERM) treatments (for example, tamoxifen) in estrogen receptor (ER)-positive breast cancer or monoclonal antibodies (for example, trastuzumab) in HER2/Neu-positive breast cancer. However, about 10% to 15% of breast cancers do not express ER, progesterone receptor (PR), and HER2/Neu receptor [1,2]. This subgroup, the so-called triple-negative category, is associated with poor prognosis because of its resistance to therapy. Its management includes the use of standard treatment such as platinum-based therapy, anthracycline, and taxanes; nevertheless, it is frequently associated with local and systemic relapse [2]. Therefore, a critical problem in the clinical strategies for the management of breast cancer is the development of molecules with effective activity in the treatments of hormone-responsive as well as triple-negative tumors. Several clinical trials assessing various therapeutic options, including the use of inhibitors of specific molecular targets such as poly-(ADP-ribose)-polymerase (PARPs) or the mammalian target of rapamycin (mTOR), used as monotherapy or combined with traditional chemotherapy, are currently ongoing [1]. Owing to their implication in several cell responses such as regulation of cell growth, survival, and apoptosis, phosphatidylinositol 3-kinase (PI3K) and the downstream Akt/mTOR pathway represent potential targets for treatment of triple-negative breast cancer [2,3].

Cruciferous vegetable consumption has been associated with lower cancer risk in several epidemiological and dietary studies [4-6]. The chemopreventive properties of these vegetables are attributed to the antitumor activity of indole-3-carbinol (I3C) and its metabolic derivatives, which have shown great potential for both prevention and treatment of cancer through numerous mechanisms such as induction of apoptosis and cell cycle arrest, antiestrogenic activity, gene expression modulation, and prevention of carcinogen-DNA adduct formation [7,8]. It has also been reported that I3C and its major condensation product 3,3'-DIM inactivate the Akt signaling pathway in breast cancer cells [9-11]. Nevertheless, the development of I3C as a therapeutic agent is limited by several factors such as its easy conversion into many polymeric products *in vivo *[12]. These compounds have some common targets but have also been demonstrated to have distinct biological effects on breast cancer cells [13,14] and the relatively high concentrations necessary to inhibit the expression of CDK6 and to induce cell cycle arrest in breast cancer (from 50 to 200 μM) [15,16].

**Figure 1:**
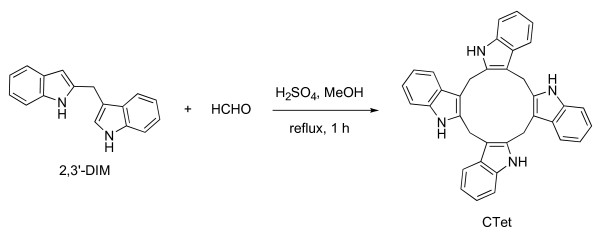
**Synthesis of CTet**. Homo-coupling of 2,3'-DIM in the presence of formaldehyde in acidic conditions is shown. Methanol was the solvent, and the mixture was refluxed for 1 hour in the dark. CTet, indole-3-carbinol cyclic tetrameric derivative.

As alternatives to I3C as a chemotherapeutic agent for the treatment of breast cancer, several I3C derivatives characterized by higher antiproliferative properties have recently been proposed [7,17-19]. I3C cyclic tetrameric derivative CTet (5,6,11,12,17,18,23,24-octahydrocyclododeca[1,2-*b*:4,5-*b'*:7,8-*b"*:10,11-*b'"*]tetraindole) (Figure 1) is an anticancer molecule that has been shown to exert interesting antiproliferative activity in both MCF-7 and MDA-MB-231 breast cancer cell lines [20]. Lucarini and colleagues [21] have optimized a straightforward, reproducible, and scalable CTet synthesis. Moreover, to improve bioavailability, they have optimized a formulation that is based on gamma-cyclodextrin (γ-CD) aqueous solution and that is about 10-fold more active with respect to the first preparation [20].

In this study, we analyze the biological responses in terms of cell cycle perturbations and autophagy induction in both ER-positive (MCF-7) and triple-negative (MDA-MB-231) breast cancer cell lines exposed to CTet obtained by a new synthetic procedure. We also characterize the molecular mechanisms leading to the inhibition of cell proliferation by using microarray-based gene expression analysis. We identified the overexpression of p21/CDKN1A (cyclin-dependent kinase inhibitor 1A) as the strongest molecular event induced by CTet treatment; the inhibition of Akt activity, revealed in CTet-treated cells, could be responsible for p21/CDKN1A overexpression in MCF-7 and p53-mutant MDA-MB-231 cells. Finally, the toxicity and antitumoral efficacy of the γ-CD-formulated CTet, obtained in a preliminary xenograft study, are discussed.

## Materials and methods

### Chemistry

All reagents, with the exception of γ-CD (CAVAMAX^® ^W8; Wacker, Munich, Germany), were purchased from Sigma-Aldrich (St. Louis, MO, USA) or Carlo Erba (Rodano, Italy) and were the highest quality reagents that were commercially available. Solvents were Carlo Erba analytic grade. Melting point, high-performance liquid chromatography/mass spectrometry (HPLC/MS), ^1^H-NMR, and ^13^C-NMR were determined as in [21]. Purification of the crude material was carried out as in [21]. Thin layer chromatography analyses were performed as in [21].

### Synthesis of CTet

To a solution of 3-[(1*H*-indol-2-yl)methyl]-1*H*-indole (2,3'-DIM) [22] (0.246 g, 1 mmol) and 37% aqueous formaldehyde (0.122 mL, 1.46 mmol) in methanol (10 mL), 96% sulphuric acid (0.063 mL) was added, and the mixture was refluxed in the dark for 1 hour. After cooling, the purple mixture was concentrated *in vacuo *in the dark. Purification of the resulting deep-purple solid by two short and fast aluminum oxide column chromatographies (cyclohexane/EtOAc 8:2) that were protected from light gave a white solid consisting (HPLC/MS) of a 2:1 mixture of CTr (5,6,11,12,17,18-esahydrocyclonona[1,2-*b*:4,5-*b'*:7,8-*b"*]triindole) and CTet (yield: 45%, 0.117 g), which was recrystallized from acetone (12 mL). CTet was obtained as a pure white solid. CTet yield was 16% (0.041 g). Melting point, ^1^H-NMR, and ^13^C-NMR are in accordance with the literature [21].

### Cell culture

The human breast carcinoma ER^+ ^(MCF-7, BT-474) and triple-negative (MDA-MB-231, BT-20) cell lines were cultured in Dulbecco's modified Eagle's medium supplemented with 10% fetal calf serum, 2 mM L-glutamine, 10 g/L non-essential amino acid, 50 mg/L streptomycin, 1,000 U/L penicillin, and 10 mg/L insulin (in MCF-7 cells) at 37°C in a humidified incubator with 5% CO_2_. For the *in vivo *experiments, MCF-7 cells were cultured in complete medium supplemented with 1 nM β-estradiol 17-cypionate for 2 weeks. All cell culture materials were purchased from Sigma-Aldrich. CTet was formulated with aqueous γ-CD solution as reported by Lucarini and colleagues [21]. In all experiments, 10 μL of the concentrated agent was added to 1 mL of cell culture medium (vehicle control, 10 μL of aqueous solution of γ-CD).

### Cell treatments

Cell proliferation was evaluated by using a [^3^H]thymidine (Sigma-Aldrich) incorporation assay. Cells were seeded at a density of 30,000 per well in 24-well tissue culture plates and allowed to attach overnight. Duplicate samples were treated for 72 hours with increasing concentrations of CTet (from 0.5 to 8.0 μM). During the last 5 hours of treatment, cells were pulsed with 3 μCi/well of [^3^H]thymidine (25 Ci/mmol) and processed as reported by Brandi and colleagues [20]. Briefly, cells were washed three times with ice-cold trichloroacetic acid (10% wt/vol) and lysed with 300 μL of 0.3 N NaOH. Aliquots (150 μL) of lysate were transferred into scintillation vials and processed for liquid scintillation counting. The results are expressed as the percentage of average count-per-minute value in drug-treated samples compared with control samples.

In a set of experiments, one batch of CTet was suspended in pure ethanol and aliquoted to evaluate the activity in different storing conditions. One aliquot was diluted in γ-CD and immediately tested in the antiproliferative assay. Three aliquots were stored at the following conditions: (a) room temperature and exposed to light, (b) room temperature and protected from light, and (c) +4°C and protected from light. Three other aliquots were diluted 1:10 in aqueous solutions of γ-CD and stored at the same conditions. The antiproliferative activity was evaluated in MCF-7 cells by using a [^3^H]thymidine incorporation assay at different time points up to 1 year.

For the gene expression, immunoblot, and cell cycle analyses, breast cancer cells were plated in six-well culture plates at a density of 150,000 cells per well and were cultured overnight. Cellular treatments were conducted at increasing concentrations of CTet or vehicle control for 24 and 48 hours. Cell survival was then evaluated by trypan blue dye exclusion assay, and after washing in phosphate-buffered saline (PBS), the cells were pelletted by centrifugation and immediately used for cell cycle analysis or stored at -20°C (for successive immunoblot or gene expression analyses). Cellular pellets prepared for gene expression analysis were stored with 300 μL of RNA-later (Sigma-Aldrich).

### High-performance liquid chromatography analyses

Quantitative determinations of CTet were performed by using an HPLC-UV method (JASCO Model PU-980). The compound was separated at room temperature on a Tracer Excel 120 ODSA 5 μm 15 × 0.46 column protected by a guard column (Pelliguard LC-18, 20 mm × 4.6 mm internal diameter, 40 μm); columns were from Teknokroma (Barcelona, Spain). CTet was quantified by UV detection at 280 nm. The volume injected was 50 μL. The mobile phase consisted of two eluents: 100% H_2_O (buffer A) and 100% acetonitrile (buffer B). CTet was eluted at a flow rate of 1.0 mL/minute and the following steady gradient program: 100% buffer A for 3 minutes, taken to 40% buffer B over the next 12 minutes, and rising to 80% buffer B from 15 to 25 minutes. This condition was held for 5 minutes, and the gradient was returned to 100% buffer A in 5 minutes.

### Cell cycle analysis

Cell cycle was analyzed by means of the propidium iodide staining procedure previously reported [23]. Briefly, cells were fixed in ice-cold 70% ethanol solution (16 hours at +4°C) and stained in propidium iodide solution (0.1% sodium citrate, 0.1% Triton X-100, 250 μg/mL RNase A, and 50 μg/mL propidium iodide). Cytofluorimetric acquisitions and sample analysis were performed with a Partec PAS flow cytometer (Partec, Münster, Germany) and FlowJo 8.6.3 software (TreeStar, Inc., Ashland, OR, USA), respectively.

### Autophagy detection by immunofluorescence analyses

MCF-7 and MDA-MB-231 cells were grown in complete medium on glass coverslips in six-well plates. After 24 hours of attachment, cells were treated with CTet for 4, 8, 12, and 24 hours. At the end of each treatment, cells were fixed with PBS (containing 4% formaldehyde) for 15 minutes and permeabilized with methanol/acetone solution for 15 minutes at room temperature. To assess the co-localization of LC3b (microtubule-associated protein 1 light chain 3) with the lysosome marker LAMP2a (lysosome-associated membrane protein type 2a), cells were incubated with a mixture containing the anti-LC3b (Sigma-Aldrich) and LAMP2a (Abcam, Cambridge, UK) primary antibodies, washed, and then probed with goat anti-rabbit AlexaFluor498 and goat anti-mouse AlexaFluor594 secondary antibodies. Nuclei were counterstained with 0.1 μg/mL 4'-6-diamidino-2-phenylindole (DAPI). Images were acquired by a Nikon Eclipse E600 microscope (Nikon Corporation, Tokyo, Japan) with ACT-1 software and were processed with an Adobe Photoshop Image Reader 7.0 (Adobe Systems Incorporated, San Jose, CA, USA).

### Gene expression analysis

#### RNA extraction and microarray analysis

Whole genome microarray analysis was performed on a CodeLink Expression Bioarray System (GE Healthcare, Piscataway, NJ, USA) on either MCF-7 or MDA-MB-231 cells treated with 6 and 12 μM CTet for 24 hours. Total RNA was purified from treated and control cells by using an RNeasy plus kit (Qiagen, Hilden, Germany). The RNA was quantified spectrophotometrically by using a Nanodrop ND-1000 (Thermo Fisher Scientific, Waltham, MA, USA); RNA integrity was evaluated on an Experion automated gel electrophoresis system (Bio-Rad Laboratories, Inc., Hercules, CA, USA). Biotin-labeled cRNA was synthesized by using a CodeLink iExpress Assay reagent kit (GE Healthcare) in accordance with the protocols of the manufacturer. Biotin-labeled cRNA obtained from each control or treated biological sample was fragmented and hybridized against three independent arrays (10 μg each) at 37°C for 22 hours (that is, three replicates for each biological sample). After hybridization, the arrays were washed, stained with Cy5-streptavidin, and scanned with a ScanArray GX scanner (PerkinElmer, Waltham, MA, USA) with a resolution of 5 μm.

The image files generated by the scanner were processed with CodeLink Expression Analysis software (GE Healthcare). Normalized data from the CodeLink software package were analyzed with GeneSifter software (Geospiza, Inc., Seattle, WA, USA) [24] for statistical validation and data mining. This comprehensive software also generated gene ontology (GO) and z-score reports. The z-score is useful for ranking GO terms by their relative amounts of gene expression changes. Positive z-scores indicate GO terms with a number of differentially expressed genes higher than expected by chance, whereas negative z-scores indicate GO terms with a number of differentially expressed genes lower than expected by chance [25]. The whole data set obtained from the two experiments on MCF-7 and MDA-MB-231 cells, both including technical replicates for each control and treated sample, was subjected to analysis of variance (ANOVA) and 5% false discovery rate calculation [26]. The cutoff parameters for differential gene expression were *P *value of 0.01 and fold change threshold of 2. Microarray data are available in the MIAME (minimum information about a microarray experiment)-compliant ArrayExpress database [27] (accession number [ArrayExpress:E-MEXP-2989]).

#### Quantitative real-time polymerase chain reaction

Real-time polymerase chain reaction (RT-PCR) was used to validate the gene expression profiles observed in the CodeLink microarray experiments. cDNA was synthesized from the same total RNA used for microarray experiments, and the SuperScript First Strand Synthesis System for RT-PCR (Invitrogen Corporation, Carlsbad, CA, USA) with oligo-dT priming was used. Primers for amplification of *p27/CDKN1B *were p27F 5'-GCAGGAATAAGGAAGCGACCT-3' and p27R 5'-TCCACAGAACCGGCATTTG-3', whereas primers for the amplification of *p21/CDKN1A *and *GADD45A *(growth arrest and DNA-damage-inducible protein alpha), together with primers for the amplification of housekeeping genes *ACTB *(actin-β) and *GAPDH *(glyceraldehyde-3-phosphate dehydrogenase), have been described elsewhere [23]. All primer pairs spanned an intron to avoid amplification of contaminating genomic DNA. RT-PCRs were performed in triplicate in a final volume of 25 μL by using SYBR green PCR master mix (Applied Biosystems, Foster City, CA, USA) with 200 nM primers in a RotorGene 6000 instrument (Corbett Life Science, Sydney, Australia). The cycling protocol was 95°C for 10 minutes followed by 40 cycles at 95°C for 10 seconds and 60°C for 45 seconds. At the end of each run, a melting curve analysis from 55°C to 90°C was performed to ensure the absence of primer dimers or non-specific products. Fold changes were calculated by using the comparative quantification application of the RotorGene 6000 software. RT-PCR-based gene expression analysis was also repeated on two new sets of biological samples, from both MCF-7 and MDA-MB-231 cells.

### Immunoblot analysis

Untreated and CTet-treated cells were lysed for 20 minutes on ice with 20 mM HEPES (pH 7.9), 25% glycerol, 0.42 M NaCl, 0.2 mM EDTA, 1.5 mM MgCl_2_, 0.5% Nonidet P-40, and 1× Complete protease inhibitor cocktail (Roche Diagnostics Ltd., Mannheim, Germany). Cell lysate was frozen and thawed twice and clarified by centrifugation at 12,000 revolutions per minute (rpm) for 10 minutes at 4°C. The subcellular fraction was obtained as follows: cells were lysed for 10 minutes on ice with 10 mM HEPES (pH 7.9), 1.5 mM MgCl_2_, 10 mM KCl, 1 mM EDTA, 1 mM Na_3_VO_4_, 1 mM NaF, 1 mM DTT, 0.1% Nonidet P-40, and 1× Complete protease inhibitor cocktail. Samples were then centrifuged at 12,000 rpm for 10 minutes at 4°C to obtain the cytosolic fraction (supernatant); the pellet was resuspended in 20 mM HEPES (pH 7.9), 25% glycerol, 0.42 M NaCl, 0.2 mM EDTA, 1.5 mM MgCl_2_, 1 mM Na_3_VO_4_, 1 mM NaF, 1 mM DTT, and 1× Complete protease inhibitor cocktail, incubated 20 minutes on ice, and centrifuged at 12,000 rpm for 10 minutes at 4°C to obtain the nuclear fraction (supernatant).

Proteins extracted were fractionated on 12% (p27 and p21) and 7.5% (Akt, phospho-Akt, and FOXO3a) SDS-PAGE and then electrically transferred to Trans-Blot transfer medium (0.2 μm) nitrocellulose membrane (Bio-Rad Laboratories, Inc.). Blots were incubated with anti-p27 (1:500) and anti-p21 (1:200) antibodies purchased from Santa Cruz Biotechnology, Inc. (Santa Cruz, CA, USA), anti-Akt and anti-phospho-Akt(Ser473) antibodies purchased from Cell Signaling Technology (Danvers, MA, USA), and anti-FKHRL1/FOXO3a (1:1,000) antibody purchased from Upstate (now part of Millipore Corporation, Billerica, MA, USA) overnight at 4°C and then 1 hour at room temperature with peroxidase-conjugated secondary antibody. Blots were treated with enhanced chemiluminescence reagents, and all of the proteins were detected and quantitated by ChemiDoc System (Bio-Rad Laboratories, Inc.). Equal protein loading was confirmed by the level of actin protein present in the membrane tested with anti-actin antibody 1:500 (Sigma-Aldrich).

### *In vivo *tumor growth inhibition

Housing and treatment of mice were in compliance with the *Guide for the Care and Use of Laboratory Animals *by Ministero della Sanità D.L. 116 (1992) and approved by the university committee for animal experiments. Female athymic Crl:CD-1-nu/nuBR nude mice (4 weeks of age) (Charles River Laboratories, Milan, Italy) were housed under pathogen-free conditions. The mice were acclimated for 1 week.

Beta-estradiol 17-cypionate (Sigma-Aldrich) was intramuscularly injected at 3 mg/kg 1 week before MCF-7 cells were transplanted into the animal and then once weekly for the duration of the experiment to support the growth of the estrogen-dependent MCF-7 tumors. The cells were inoculated subcutaneously at 1.1 × 10^6 ^cells per inoculum on one flank in a final volume of 200 μL containing 100 μL of Matrigel (BD Biosciences, San Jose, CA, USA) and 100 μL of cells suspended in 0.9% NaCl.

Twenty days after the cell inoculation, the mice received the CTet intraperitoneally at the concentration of 5 mg/kg per day for 5 days per week for a total of 2 weeks. The mice in the control (placebo) group received the same volume of the vehicle as the CTet-treated mice. At least four animals were studied for each experimental group. Each xenograft was monitored by using a calliper to externally measure tumors in two dimensions. Tumor volume (in cubic millimeters) was calculated as *a*^2 ^× *b *× 0.5, where *a *is the length and *b *is the width of the tumor.

### Statistical analyses

Data are expressed as mean ± standard error of the mean of at least three separate experiments. The half inhibitory concentration (IC_50_) values of antiproliferative activity were calculated by nonlinear regression by using the equation y = 100 - Top·x ^(HillSlope)^/(IC_50_^(HillSlope) ^+ x^(HillSlope)^) (Prism5; GraphPad Software, Inc., La Jolla, CA, USA). Statistical analysis was performed by using the Mann-Whitney test or one-way ANOVA followed by Tukey *post hoc *test as appropriate (Prism5).

## Results

### Synthesis of CTet

The protocol herein reported to obtain CTet was optimized in terms of reagents, temperature, and time. The developed method proved to be reproducible with regard to CTet/CTr ratio and yield (Additional file 1, CTr formation).

### Antiproliferative activity of CTet

**Figure 2:**
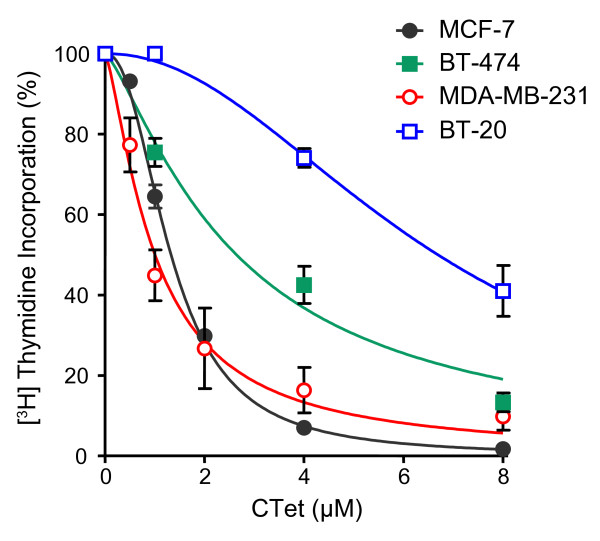
**Antiproliferative activity of the CTet formulated in γ-cyclodextrin aqueous solution**. Estrogen receptor-positive (MCF-7 and BT-474) and triple-negative (MDA-MB-231 and BT-20) human breast cancer cell lines were exposed to increased concentrations of CTet. Cell proliferation was evaluated by [^3^H]thymidine incorporation into cellular DNA after 72 hours of treatment. Results are shown as the percentage of [^3^H]thymidine incorporation in treated cells compared with control cells (vehicle only). Data are expressed as mean ± standard error of the mean of at least three separate experiments. CTet, indole-3-carbinol cyclic tetrameric derivative.

It has been previously reported that CTet formulated with γ-CD is able to inhibit proliferation of both MCF-7 and MDA-MB-231 breast cancer cell lines [21]. In this study, we confirm the dose-dependent activity of CTet in both MCF-7 (IC_50 _= 1.32 ± 0.03 μM) and MDA-MB-231 (IC_50 _= 1.00 ± 0.01 μM). In addition, we investigated the drug effect on DNA synthesis in two other breast cancer cell lines: BT474 (ER^+^) and BT-20 (triple-negative) (Figure 2). The IC_50 _values obtained in the two cell lines were 2.64 ± 0.28 and 6.69 ± 0.37 μM, respectively.

### Analysis of cell cycle perturbations

**Figure 3:**
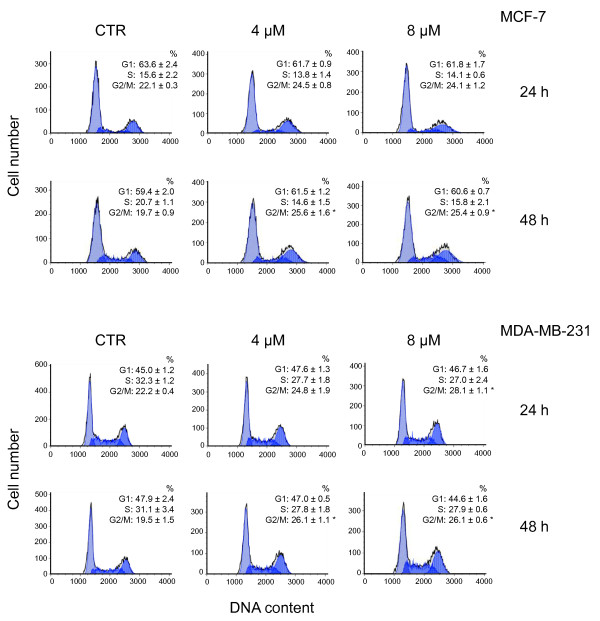
**Cell cycle effects of the CTet in MCF-7 and MDA-MB-231 cell lines**. DNA content profiles of cells that were exposed for 24 and 48 hours to 4 and 8 μM CTet or γ-cyclodextrin solution (CTR), stained with propidium iodide, and analyzed by flow cytometry are shown. Data are from one representative experiment. The percentages of cells in the different phases of the cell cycle are presented as the mean of three experiments ± standard error of the mean. Asterisks indicate statistically significant values with respect to CTR (one-way analysis of variance followed by Tukey *post hoc *test; *P *< 0.05). CTet, indole-3-carbinol cyclic tetrameric derivative.

Cell cycle analysis was carried out in MCF-7 and MDA-MB-231 to evaluate the effect of CTet on cell cycle progression. Cells were treated with CTet for 24 and 48 hours at the final concentrations of 4.0 and 8.0 μM and then stained with propidium iodide for flow cytometric analyses. Results showed that CTet induced G_2_/M accumulation in both MCF-7 and MDA-MB-231 cells (Figure 3). After 48 hours of treatment, the cellular population in the G_2_/M phase significantly increased from 19.7% ± 0.9% to 25.4% ± 0.9% in MCF-7 and from 19.5% ± 1.5% to 26.1% ± 0.6% in MDA-MB-231 (*P *< 0.05).

### CTet-treated MDA-MB-231 cells show morphological features of autophagy

**Figure 4:**
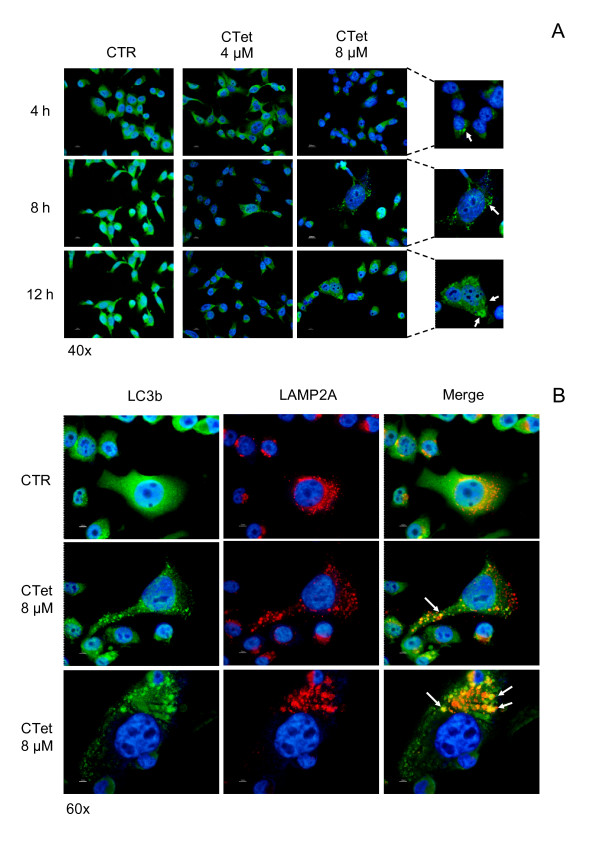
**Morphological features of autophagy in CTet-treated MDA-MB-231 cells**. Autophagy induction in MDA-MB-231 cells following exposure to CTet was detected by recruitment of LC3b protein to autophagosomes **(a) **and fusion between autophagosomes and lysosomes in terms of co-localization of LC3b and the lysosome marker LAMP2a **(b)**. CTet, indole-3-carbinol cyclic tetrameric derivative; CTR, control; LAMP2a, lysosome-associated membrane protein type 2a; LC3b, microtubule-associated protein 1 light chain 3.

Although the exposure of breast cancer cells to CTet failed to induce apoptosis, specific morphological features of autophagy were detectable in drug-treated MDA-MB-213 cells. In particular, in cells exposed at different time points to 8.0 μM CTet, the occurrence of autophagy was assessed by immunofluorescence analysis of LC3b protein. The LC3b protein is recruited to the autophagosome membrane during the autophagy process; consequently, changes in the intracellular localization of LC3b provide a reliable molecular marker for the detection of autophagy. A significant increase in the percentage of MDA-MB-231 cells with a characteristic punctate pattern of LC3b expression was appreciable following exposure to CTet (Figure 4a). Fusion between autophagosomes and lysosomes also represents an important regulatory step of autophagy pathway and can be monitored by co-localization of LC3b and lysosome markers LAMP1 or LAMP2a. In MDA-MB-231 cells, autophagic vescicles were found to co-localize with lysosome after exposure to CTet, as demonstrated by the overlapping of LC3b and LAMP2a signals in combined immunofluorescence experiments (Figure 4b; Additional file 2, Figure S1). Taken together, these results indicate that autophagic lysosomal activity is significantly upregulated in MDA-MB-231 as a consequence of CTet exposure. Autophagy features have been transiently observed also in MCF-7 cells following exposure to the highest CTet concentration (data not shown).

### Changes of gene expression profile induced by CTet in MCF-7 and MDA-MB-231 cell lines

The molecular mechanisms involved in CTet response in MCF-7 and MDA-MB-231 cells lines were investigated by means of microarray technology. Both cell lines were treated with 6.0 and 12.0 μM CTet for 24 hours before cell harvesting. This early time point was chosen with the aim of observing the changes in gene expression before they have an effect on cell metabolism. Since the antiproliferative activity and biological responses of CTet were similar among cell lines and independent from the hormonal receptor status, the genes sharing a common expression pattern in both MCF-7 and MDA-MB-231 cell lines, in either 6.0 or 12.0 μM CTet treatment conditions, were selected with GeneSifter software.

First, when a differential expression cutoff of 2 (ANOVA, Benjamini-Hochberg false discovery rate correction, *P *< 0.01) was used, a total of 960 genes differentially expressed in at least one treatment in MCF-7 or MDA-MB-231 cells (or both) were identified. Then, a further analysis of the expression pattern in this subset of genes (Pearson uncentered correlation coefficient of 0.98) revealed that 116 genes were upregulated (search pattern: control = 1; 6.0 μM >1; 12.0 μM >2) and 177 genes were downregulated (search pattern: control = 1; 6.0 μM <1; 12.0 μM < 0.5) in both cell lines (Additional files 3 and 4, Tables S1 and S2, respectively). To explore the biological significance of the transcriptome response shared by the two cell lines, z-score reports containing the most significant GO terms were generated from the commonly upregulated (Table 1) and downregulated (Table 2) genes. Criteria for selection were GO terms containing at least 20 genes, number of genes differentially expressed within the assigned ontology of at least 3, and z-score of at least 4 or not more than -4. The ontology list was then pruned by hand for related GO terms to remove any over-represented branches of the GO hierarchy. When both a parent and a child term were present on the list, the parent term was removed if its presence was due entirely to genes meeting the criterion for the child term.

**Table 1:** Selected ontologies of commonly upregulated genes

Term	**Genes**^**a**^	Gene set on the array	Z-score
Biological process			
Response to stimulus	39	3,137	4.82
Response to stress	27	1,753	5.05
Response to unfolded protein^b^	3	62	4.14
Acute inflammatory response^b^	5	99	5.5
Acute-phase response^b^	4	43	7.11
Regulation of acute inflammatory response^b^	3	26	6.95
Response to chemical stimulus	21	1,396	4.26
Response to organic substance^b^	15	845	4.25
Response to hormone stimulus^b^	10	440	4.35
Response to steroid hormone stimulus^b^	7	220	4.75
Response to glucocorticoid stimulus^b^	6	94	6.98
Leukocyte chemotaxis^b^	3	60	4.23
Response to oxygen levels^b^	5	151	4.12
Response to toxin^b^	3	64	4.05
Response to extracellular stimulus	7	242	4.41
Positive regulation of response to external stimulus^b^	4	87	4.63
Cellular response to extracellular stimulus^b^	5	82	6.19
Cellular response to nutrient levels^b^	4	62	5.73
Cellular response to starvation^b^	3	43	5.2
Response to biotic stimulus	10	424	4.49
Response to virus^b^	6	147	5.24
Cell death	24	1,227	6.04
Apoptosis	22	1,116	5.8
Regulation of cell death^b^	17	872	4.99
Regulation of apoptosis^b^	16	859	4.62
Negative regulation of cell death^b^	10	402	4.69
Negative regulation of apoptosis^b^	9	391	4.16
Induction of apoptosis^b^	8	333	4.06
Anti-apoptosis^b^	8	226	5.49
Positive regulation of anti-apoptosis^b^	3	32	6.18
Cellular component disassembly involved in apoptosis^b^	3	27	6.81
Release of cytochrome *c *from mitochondria^b^	3	24	7.27
Protein folding	6	158	4.99
Leukocyte migration	4	86	4.66
Autophagy	3	48	4.86
Regulation of synaptic plasticity	3	57	4.37
Iron ion homeostasis	4	31	8.54
Positive regulation of smooth muscle cell proliferation	3	28	6.67
Regulation of viral reproduction	3	28	6.67
Cellular component			
Integral to membrane	9	4,459	-4.27
Autophagic vacuole	3	20	8.2
Molecular functions			
Heat-shock protein binding	5	71	7.14
Unfolded protein binding	5	103	5.69
Antioxidant activity	3	45	5.35

^a^Number of genes differentially expressed within the assigned ontology. ^b^Subcategory within a given ontology.

**Table 2:** Selected ontologies of commonly downregulated genes

Term	**Genes**^**a**^	Gene set on the array	Z-score
Biological process			
Cell cycle	20	957	4.42
Spindle organization^b^	4	54	5.31
DNA metabolic process	18	559	6.32
DNA replication^b^	13	228	8.13
DNA-dependent DNA replication^b^	5	71	5.75
DNA-dependent DNA replication initiation^b^	3	25	6.13
Cellular component			
Chromosomal part	13	381	5.57
Condensed chromosome kinetochore^b^	4	69	4.52
Replication fork^b^	3	33	5.2
Molecular function			
Transmembrane receptor protein tyrosine kinase activity	4	64	4.81
DNA helicase activity	3	41	4.6
Insulin-like growth factor binding	3	24	6.32

^a^Number of genes differentially expressed within the assigned ontology. ^b^Subcategory within a given ontology.

Concerning the upregulated genes, the main terms in the biological process category included 'response to stimulus' (stress, chemical stimulus, extracellular stimulus, and so on), 'apoptosis', 'protein folding', and 'autophagy'. The cellular components were linked to 'autophagic vacuole' and 'integral to membrane', the latter having a number of differentially expressed genes lower than expected by chance (negative z-score; Materials and methods). In the molecular function category, transcripts were linked to 'heat shock protein binding', 'unfolded protein binding', and 'antioxidant activity' (Table 1).

With respect to downregulated genes, the biological process category included 'cell cycle' and 'DNA metabolic process', whereas the cellular components were linked to 'chromosomal part'. Finally, the molecular functions category included 'transmembrane receptor protein tyrosine kinase activity', 'DNA helicase activity', and 'insulin-like growth factor binding' (Table 2).

**Figure 5:**
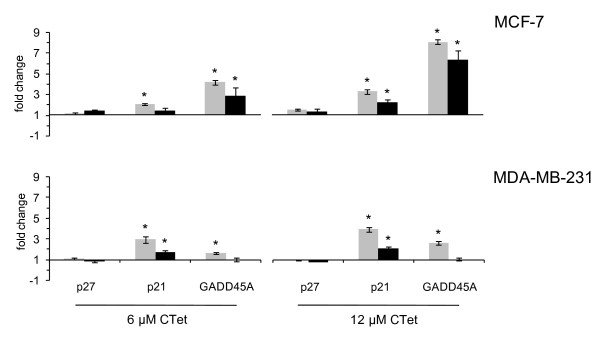
**Gene expression analysis of p27, p21, and GADD45A**. Quantitative real-time polymerase chain reaction (gray bars) and microarray-based (black bars) expression analyses of p27, p21, and GADD45A genes were carried out in MCF-7 and MDA-MB-231 cell lines treated with CTet 6.0 μM (left) and 12.0 μM (right) for 24 hours. For quantitative real-time polymerase chain reaction, GAPDH was used as a housekeeping gene. Data are shown as mean ± standard deviation. Asterisks indicate statistically significant values (*P *< 0.01). GADD45A, growth arrest and DNA-damage-inducible protein alpha; GAPDH, glyceraldehyde-3-phosphate dehydrogenase.

To further verify the microarray transcription profile results, selected genes were analyzed by quantitative RT-PCR. For these analyses, three genes associated with cell cycle arrest were selected: *p27/CDKN1B *(already identified by using CTet previously prepared [20]), *GADD45A*, and *p21/CDKN1A *(identified as γ-CD-formulated CTet-induced genes in the microarray experiments). *GAPDH *was used as a housekeeping gene. Actin-β was used in a subset of samples (not shown) as an alternative housekeeping gene to confirm the results obtained with *GAPDH*. The results (Figure 5) were almost superimposable with those of the microarray experiments, although a greater sensitivity of RT-PCR compared with microarray analysis was revealed. *p27/CDKN1B *gene expression appeared unchanged, whereas *p21/CDKN1A *showed upregulation in all conditions tested in both cell lines. Moreover, a significant upregulation of *GADD45A *gene was observed in both MCF-7 and MDA-MB-231 cell lines, although the microarray experiment did not show any significant upregulation in the latter cell line. The quantitative RT-PCR analysis has also been performed on two new sets of biological samples, from either MCF-7 or MDA-MB-231 cells, confirming the upregulation of *p21/CDKN1A *and *GADD45A*. The average data obtained from three biological samples are shown in Figure S2 of Additional file 5.

### Immunoblot analysis

**Figure 6:**
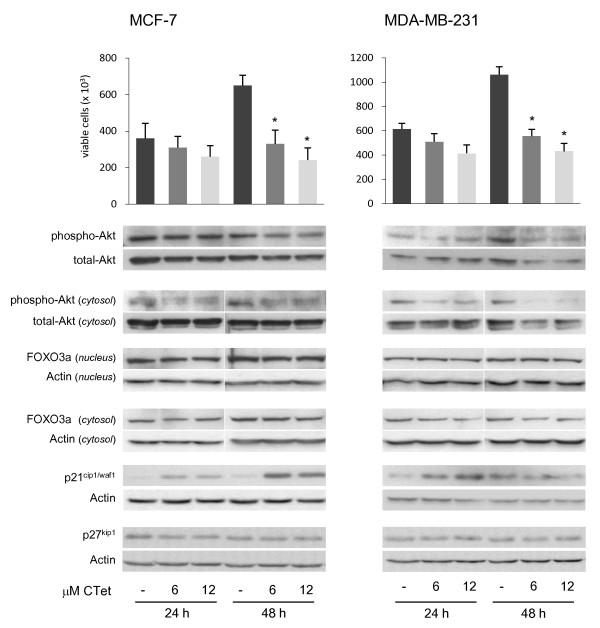
**Cell viability and immunoblot analyses in CTet-treated MCF-7 and MDA-MB-231 breast cancer cell lines**. Cells were treated for 24 and 48 hours with 6.0 and 12.0 μM CTet or vehicle only (γ-cyclodextrin aqueous solution). After treatments, cell viability was evaluated by Trypan blue dye exclusion assay and cell extracts were processed as described in the Materials and methods ('Immunoblot analysis'). Akt activity was analyzed by using a phospho-sensitive Akt antibody in total cell extracts and in cytosolic cellular fractions. FOXO3a localization was evaluated by separation of nuclear and cytosolic proteins, and p21 and p27 overexpression was evaluated in total cell extracts. FOXO3a, p27, and p21 were normalized to actin, and phospho-Akt was normalized to total-Akt. Cell counts are presented as the mean ± standard error of the mean of three separate experiments. Asterisks indicate statistically significant values with respect to untreated cells (one-way analysis of variance followed by Tukey *post hoc *test; *P *< 0.01). Blots are representative of at least two separate experiments. CTet, indole-3-carbinol cyclic tetrameric derivative.

The significant upregulation of *p21/CDKN1A *CDKs inhibitor gene, monitored by microarray studies, was further investigated at the protein level by immunoblot analysis. The results showed that p21/CDKN1A was overexpressed in both MCF-7 and MDA-MB-231 cell lines after 24 hours of treatment (>7-fold to actin) and in MCF-7 cells after 48 hours of treatment (>9-fold to actin) (Figure 6). Unlike in previous results [20], CTet formulated did not induce p27^kip1 ^(cyclin-dependent kinase inhibitor 1B) overexpression in MCF- 7 and MDA-MB-231 cells. Further investigations were therefore directed to establish the involvement of FOXO3a transcription factor localization and Akt activity, both of which are involved in p21/CDKN1A expression [28,29]. To evaluate the role of the PI3K/Akt pathway in p21/CDKN1A expression in CTet-treated breast cancer cell lines, Akt activity was detected by using a specific anti-phospo-Akt antibody, the phosphorylated form of Akt protein. The results showed that, in both MCF-7 and MDA-MB-231, phospho-Akt decreases after 48 hours of treatment (about 0.8-fold and 0.6-fold to total-Akt in MCF-7 and MDA-MB-231, respectively), whereas the decrement of the cytosolic fraction of phospho-Akt was observed after 24 hours of treatment (about 0.6-fold and 0.4-fold to total-Akt in MCF-7 and MDA-MB-231, respectively) (Figure 6). Akt can inactivate FOXO3a via phosphorylation and subsequent translocation to cytosol, but in CTet-treated cells, there was no evidence of variation in FOXO3a localization, suggesting that this transcription factor was not involved in the overexpression of p21/CDKN1A.

### Effect of CTet on xenograft tumor growth in athymic nude mice

**Figure 7:**
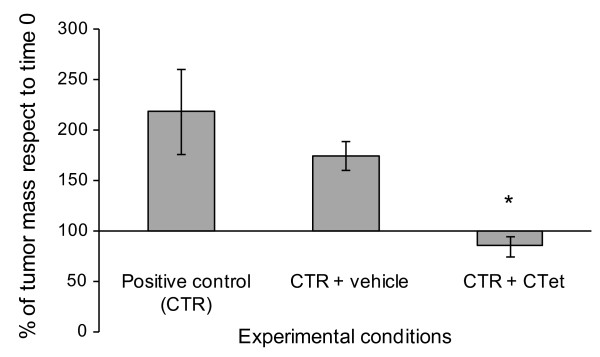
***In vivo *effects of CTet on the growth of human MCF-7 breast cancer cell-derived tumors from xenografts in athymic mice**. Athymic female mice were inoculated with MCF-7 cells and then intraperitoneally treated with CTet (5 mg/kg) or vehicle (γ-cyclodextrin solution) for 2 weeks, as described in Materials and methods. The percentage of residual tumor mass has been calculated with respect to time 0, which corresponds to the start of treatment. Positive control (CTR) refers to four mice bearing the tumor and not treated, CTR + vehicle refers to four mice bearing the tumor and receiving the vehicle, and CTR + CTet refers to five mice bearing the tumor and receiving CTet. The results are presented as mean ± standard deviation of the number of mice indicated above. The asterisk indicates statistically significant value with respect to CTR and CTR + vehicle (Mann-Whitney test; *P *< 0.05). CTet, indole-3-carbinol cyclic tetrameric derivative.

Toxicity studies aimed at establishing median lethal dose (LD50) showed that CTet in the concentration range of 0.5 to 15 mg/kg did not cause any toxic effect (data not shown). To evaluate the potential therapeutic efficacy of systemic administration of CTet, a preliminary experiment was performed in human breast cancer xenograft-bearing nude mice. The MCF-7 cells were inoculated subcutaneously on one flank of nude mice as described in Materials and methods. Twenty days after cell inoculation, intraperitoneal treatment with CTet was started at the dose of 5 mg/kg per day (five treatments a week) and lasted for 2 weeks. The results showed that the treatment blocked the increase of tumor mass, which significantly increased in the animals either not treated or receiving the vehicle (Figure 7). After 2 weeks of treatment, both mice groups receiving CTet or the vehicle did not show any alteration either in body weight or in the hematological parameters with respect to the untreated mice (data not shown).

### CTet activity in different storing conditions

CTet activity in different storing conditions was evaluated up to 1 year. Aliquots of CTet were stored at different conditions of temperature and light exposition (Additional file 6, Table S3), and the activity was evaluated in MCF-7 at different time points (4, 8, 12, and 20 weeks and after 1 year). The results showed that the antiproliferative activity, expressed as IC_50_, remains nearly unchanged up to 1 year (Additional file 6, Table S3). Chemical stability of the molecule was assessed by HPLC after 1 year of storage. The chromatographic profile showed that, in all light-protected conditions, more than 95% of the CTet maintained its chromatographic behavior, confirming the biological data; however, in non-light-protected conditions, a loss of the molecular integrity (~15%) was detected (data not shown).

## Discussion

In the last decades, the mortality rates of breast cancer have decreased [30] as a result of implementation of screening [31], improvements in the local management of early breast cancer [32], and the introduction of adjuvant systemic treatments [33]. However, breast cancer is the leading cause of cancer-related death for women in Europe and the US [34,35]. The treatment of a subgroup of breast tumors resistant to targeted therapies - named triple-negative tumors because of a lack of estrogen, progesterone, and HER2/Neu receptors - is a problem that remains unsolved [1,2].

Derivatives of I3C with good antiproliferative activity independently of hormonal receptor status have been widely studied in recent years [7,8,17-19,36]. The potential of one of these molecules, the I3C cyclic tetrameric derivative CTet, in the inhibition of breast cancer cell proliferation was shown [20]. However, further analysis revealed that, owing to the presence of other oligomeric derivatives of I3C, the synthetic method previously reported did not give a pure compound.

Recently, Lucarini and colleagues [21] proposed a straightforward synthesis of CTet, but this method, as well as the others reported in the literature [20,37,38], is not sufficiently advantageous. In this paper, we describe a new synthesis involving 2,3'-DIM homo-coupling in the presence of formaldehyde in acidic medium. This synthetic procedure gave an acceptable CTet yield, higher than those already reported [20,21,37,38].

The antiproliferative activity of CTet formulated with γ-CD, first evaluated in MCF-7 and MDA-MB-231 [21], was confirmed in this study in two additional breast cancer cell lines (BT-474 and BT-20), showing that CTet is able to inhibit cell proliferation about 10-fold more than the first formulation in all cell lines tested.

The cell cycle and molecular analyses in MCF-7 and MDA-MB-231 cells treated with formulated CTet did not show the induction of G_1 _cell cycle arrest or the overexpression of CDKs p27 inhibitor as described previously [20]. In fact, CTet inhibited cell proliferation by activating mechanisms resulting in an increased amount of viable cells in the G_2_/M phase of the cell cycle.

Autophagy is a multistep process in which cellular proteins and organelles are sequestered, delivered to lysosomes, and digested by lysosomal hydrolases. This process culminates when the nascent autophagosome fuses with the endosomal/lysosomal system to create a fully functional degradative compartment, the autolysosome. By a combined immunofluorescence approach, we were able to detect such a fusion in CTet-treated MDA-MB-231 cells as the co-localization of the autophagosome marker LC3b and the lysosomal marker LAMP2a [39]. Whether treatment-induced autophagy in these cells represents a survival mechanism or initiates a nonapoptotic cell death remains uncertain [40]. However, the evidence of a significant drug-induced antiproliferative effect in the absence of a clear activation of apoptotic pathways, as observed in gene expression analysis (see below), would suggest the possibility that MDA-MB-231 cells undergo autophagic cell death.

From gene expression analysis, a reliable list of genes upregulated or downregulated in response to CTet treatment was obtained. Interestingly, several genes involved in suppression of cell proliferation resulted upregulated (for example, *IL6*, *IL8*, *p21/CDKN1A*, and *HBP1*) whereas other genes involved in cell cycle progression were downregulated (for example, *CDK2*, *CCNE2*, *E2F2*, *MCM3*, and *PKMYT1*), recapitulating the cell cycle profile alterations observed.

The gene expression analysis revealed also the cellular response to the stress/stimulus induced by the drug treatment, through the upregulation of genes involved in oxidative stress response (for example, *HMOX1*, *TXNRD1*, and *SOD2*), xenobiotic metabolism, (for example, *CYP1B1*, *AKR1C1*, and *AHR*), response to unfolded proteins (for example, *DNAJB1*, *DNAJB4*, *DNAJB9*, and *HSPA1A*), and inflammatory response (for example, *IL6*, *CEBPB*, *CCL5*, *PTGS2*, and *CFB*). Moreover, the upregulation of either pro-apoptotic (for example, *BBC3*, *DEDD2*, and *PMAIP1*) or anti-apoptotic (for example, *BAG3 *and *BEX2*) genes do not provide evidence of any apoptosis induction. In addition, microarray results supported (from a molecular point of view) the autophagy process observed in both cell lines. In fact, the autophagy-related genes *WIPI1 *(ATG18), *GABARAPL1 *(ATG8), *MAP1LC3B *(LC3B), and *SQSTM1 *were found upregulated. Moreover, RT-PCR results, besides confirming the upregulation of *p21/CDKN1A*, showed a significant upregulation of *GADD45A *gene also in MDA-MB-231 cells.

Altogether, our results suggest that the genes responsible for the arrest of cell proliferation could be the *p21/CDKN1A *and *GADD45A*. The p21 protein is a universal inhibitor of the cyclin-dependent kinase (CDK) family [41] and is able to block cell cycle progression in either the G_1_/S or the G_2_/M phase [41-44]. GADD45A interacts with Cdc2 and inhibits its kinase activity, playing an important role in the regulation of the G_2_/M cell cycle checkpoint [45,46]. This finding suggests that CTet treatments inhibit cell cycle progression in breast cancer cells by acting on both G_1_/S and G_2_/M cell cycle checkpoints.

The upregulation of *p21/CDKN1A *and *GADD45A *has to be considered not dependent by p53 induction since p53 gene is mutated in MDA-MB-231 cells [47,48]. Moreover, a search for the important transcription factor-binding sites enriched in the selected 116 commonly upregulated genes (Table 4S) by using Distinct Regulatory Elements of co-regulated genes (DiRE) algorithm [49,50] did not show the presence of p53 among the top 50 transcription factors identified (data not shown).

The immunoblot analysis also revealed the inhibition of Akt activity in both cell lines tested. The protein kinase components of protein kinase B (Akt) pathway represent one set of potential targets for treatment of triple-negative tumors [2]. In fact, PI3K and downstream AKT/protein kinase B family members have been implicated in several cell responses, including the protection of cells from apoptosis, the promotion of cell proliferation, and different metabolic responses [3], and may also be implicated in both p53-dependent and p53-independent expression of *p21/CDKN1A *[28] and *GADD45A *[51]. The inhibition of Akt activity could then play a central role in the antitumoral properties of CTet, as well as I3C and 3,3'-DIM [9-11], and could explain, at least in part, the induction of p53-independent *p21/CDKN1A *and *GADD45A *overexpression in the p53 mutant MDA-MB-231 cell line.

The mechanisms by which CTet-induced overexpression of *p21/CDKN1A *and *GADD45A*, resulting in the inhibition of cell proliferation and autophagy, will be further investigated to establish the eventual involvement of upstream PI3K/AKT or other molecular pathways or both.

The *in vivo *biological activity of the γ-CD-formulated CTet was promising since the administration was effective in blocking the increase of tumor mass in xenograft study. Moreover, neither CTet formulation nor γ-CD aqueous solution alone showed toxicity.

CTet stored in the dark is a very stable molecule. Indeed, more than 98% of CTet formulated in γ-CD aqueous solution and stored at room temperature and protected from light retained its stability and biological activity up to 1 year (IC_50 _<1 μM) in MCF-7 cells. On the whole, the results obtained with γ-CD formulated CTet are very important because the utilization of dimethyl sulfoxide as a vehicle is not needed [17,20,36].

## Conclusions

Our results showed that CTet is able to induce G_2_/M cell accumulation and autophagic response in both hormone-responsive and triple-negative breast cancer cells. The overexpression of *p21/CDKN1A *could be the main molecular event responsible for the inhibition of cell proliferation, together with the inhibition of Akt activity and the overexpression of *GADD45A *and the autophagy-related genes. Results from the *in vivo *study also showed that CTet formulated with γ-CD is a promising and injectable anticancer agent that deserves additional studies to support the data reported here.

## Abbreviations

γ-CD: gamma-cyclodextrin; ANOVA: analysis of variance; ^13^C-NMR: carbon-13 nuclear magnetic resonance; CTet: indole-3-carbinol cyclic tetrameric derivative; CTr: indole-3-carbinol cyclic trimeric derivative; ER: estrogen receptor; *GADD45A*: growth arrest and DNA-damage-inducible protein alpha; *GAPDH*: glyceraldehyde-3-phosphate dehydrogenase; GO: gene ontology; ^1^H-NMR: proton nuclear magnetic resonance; HPLC: high-performance liquid chromatography; HPLC/MS: high-performance liquid chromatography/mass spectrometry; I3C: indole-3-carbinol; IC_50_: half inhibitory concentration; LAMP2a: lysosome-associated membrane protein type 2a; LC3b: microtubule-associated protein 1 light chain 3; mTOR: mammalian target of rapamycin; *p21/CDKN1A*: cyclin-dependent kinase inhibitor 1A; p27^Kip1^: cyclin-dependent kinase inhibitor 1B; PBS: phosphate-buffered saline; PI3K: phosphatidylinositol 3-kinase; PR: progesterone receptor; rpm: revolutions per minute; RT-PCR: real-time polymerase chain reaction.

## Competing interests

MM and GB are listed as inventors on US Patent 7,645,788 ('Tetramerous derivative of indole-3-carbinol with anti-carcinogenic activity and method of synthesis of said derivative'), held by the University of Urbino. The remaining authors declare that they have no competing interests.

## Authors' contributions

MM and GB coordinated the studies and helped to design the experiments. MDS performed cell culture experiments and immunoblot analyses and drafted the manuscript. LG carried out the gene expression analyses. SL and AD performed and optimized the synthesis of CTet. MFP and AF carried out the xenograft experiments and HPLC analyses. MF performed the cytofluorimetric analyses. CDM and NZ carried out immunofluorescence analyses for the autophagy detection. All authors helped to draft the manuscript and read and approved the final manuscript.

## Supplementary Material

Additional file 1 **CTr formation**. Plausible mechanisms of CTr formation in the CTet synthesis. Click here for file

Additional file 2 **Figure S1**. Autophagic morphological features induced by serum starvation in MDA-MB-231 cells (positive control) detected by fusion between autophagosomes and lysosomes, in terms of co-localization of LC3b and the lysosome marker LAMP2a. Click here for file

Additional file 3 **Table S1**. Transcriptome analysis was performed on MCF-7 and MDA-MB-231 cells treated with 6.0 μM and 12.0 μM CTet for 24 hours. The genes up-regulated in both MCF-7 and MDA-MB-231 cell lines, either in 6.0 μM and 12.0 μM CTet treatment conditions, were selected using GeneSifter software. The software analysis allowed to identify a list of 116 genes significantly (*p *< 0.01) up-regulated in both cell lines. Click here for file

Additional file 4 **Table S2**. Transcriptome analysis was performed on MCF-7 and MDA-MB-231 cells treated with 6.0 μM and 12.0 μM CTet for 24 hours. The genes down-regulated in both MCF-7 and MDA-MB-231 cell lines, either in 6.0 μM and 12.0 μM CTet treatment conditions, were selected using GeneSifter software. The software analysis allowed to identify a list of 177 genes significantly (*p *< 0.01) down-regulated in both cell lines. Click here for file

Additional file 5 **Figure S2**. Quantitative real-time PCR of p27, p21 and GADD45A genes were carried out in MCF-7 and MDA-MB-231 cell lines treated with CTet 6.0 μM (left) and 12.0 μM (right) for 24 hours. Data are shown as mean ± standard deviation of three separate experiments. Asterisks indicate statistically significant values (*p *< 0.01). Click here for file

Additional file 6 **Table S3**. Antiproliferative activity of CTet in different storing conditions in MCF-7 cells. Aliquots of CTet were stored at different conditions of temperature and light exposition and the activity was then evaluated in MCF-7 at different time points (4, 8, 12, 20 weeks and after one year). Results are reported as IC_50 _values. Click here for file
