# Supplementary material for: The indole-3-carbinol cyclic tetrameric derivative CTet inhibits cell proliferation via overexpression of p21/CDKN1A in both estrogen receptor-positive and triple-negative breast cancer cell lines
Source: Breast Cancer Res. 2011 Mar 24;13(2):R33. doi: 10.1186/bcr2855 (PMC3219196; doi:10.1186/bcr2855)
Supplement: Additional file 1 — CTr formation. Plausible mechanisms of CTr formation in the CTet synthesis. [file bcr2855-S1.PDF]

Plausible mechanisms of CTr formation in the CTet synthesis.

In CTet synthesis, CTr is also formed maybe because of the low stability of the starting material 2,3’-DIM. In the reaction conditions, that molecule could give a monomer indolyl fragment able to react with 2,3’-DIM to form CTr. Another plausible mechanism could involve the CTet ring opening, the elimination of a monomer indolyl fragment, and the consequent ring closure to CTr.
