# Supplementary figures and images for: The indole-3-carbinol cyclic tetrameric derivative CTet inhibits cell proliferation via overexpression of p21/CDKN1A in both estrogen receptor-positive and triple-negative breast cancer cell lines
Source: Breast Cancer Res. 2011 Mar 24;13(2):R33. doi: 10.1186/bcr2855 (PMC3219196; doi:10.1186/bcr2855)

Positive Controls

LC3b

LAMP2A

Merge

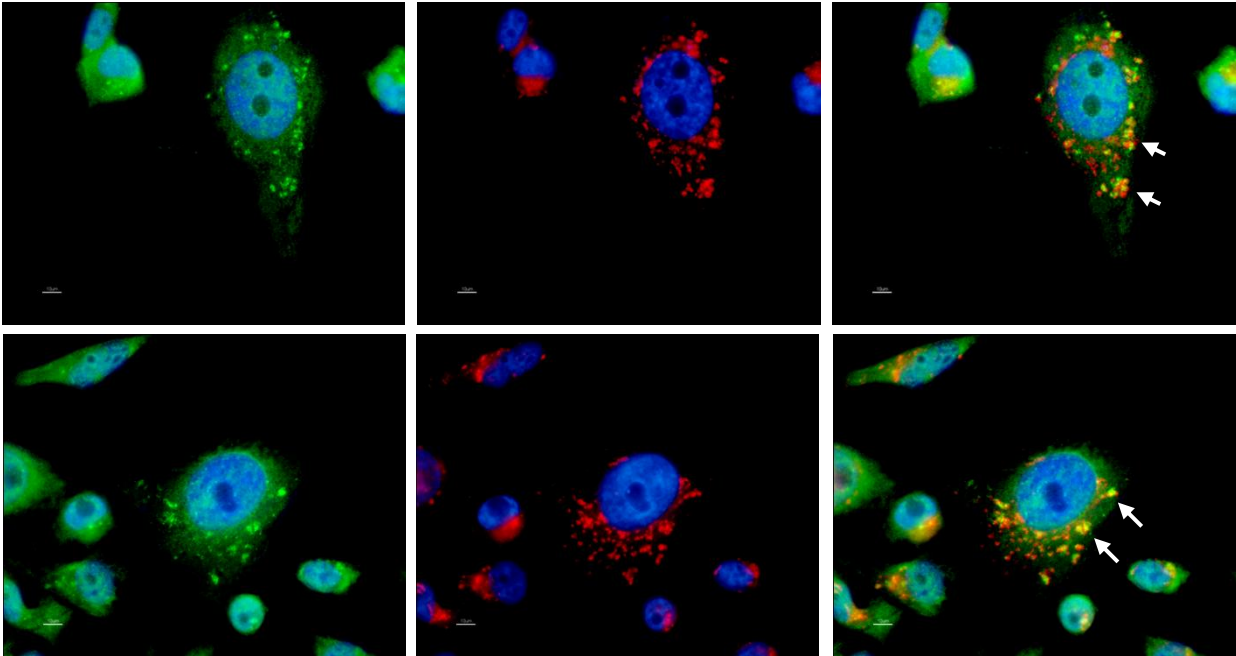

60 x

Supplement: Additional file 2 — Figure S1. Autophagic morphological features induced by serum starvation in MDA-MB-231 cells (positive control) detected by fusion between autophagosomes and lysosomes, in terms of co-localization of LC3b and the lysosome marker LAMP2a. [file bcr2855-S2.PDF]

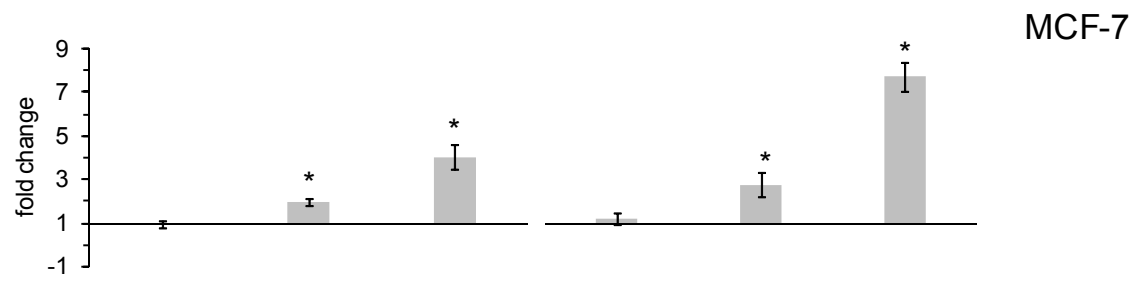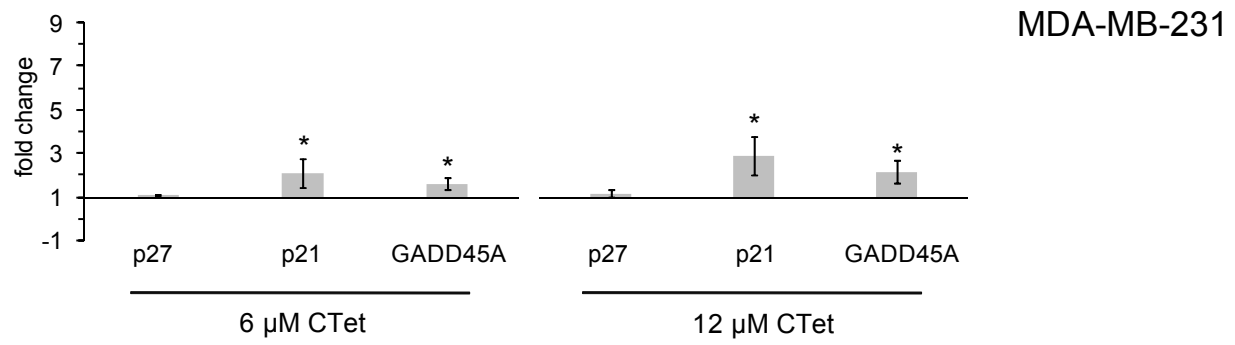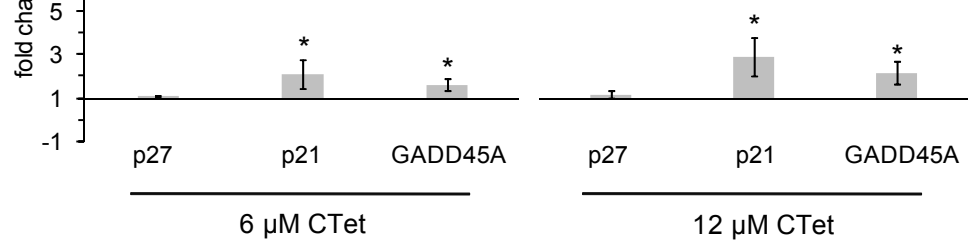

Supplement: Additional file 5 — Figure S2. Quantitative real-time PCR of p27, p21 and GADD45A genes were carried out in MCF-7 and MDA-MB-231 cell lines treated with CTet 6.0 μM (left) and 12.0 μM (right) for 24 hours. Data are shown as mean ± standard deviation of three separate experiments. Asterisks indicate statistically significant values (p < 0.01). [file bcr2855-S5.PDF]
