# Supplementary material for: The indole-3-carbinol cyclic tetrameric derivative CTet inhibits cell proliferation via overexpression of p21/CDKN1A in both estrogen receptor-positive and triple-negative breast cancer cell lines
Source: Breast Cancer Res. 2011 Mar 24;13(2):R33. doi: 10.1186/bcr2855 (PMC3219196; doi:10.1186/bcr2855)
Supplement: Additional file 3 — Table S1. Transcriptome analysis was performed on MCF-7 and MDA-MB-231 cells treated with 6.0 μM and 12.0 μM CTet for 24 hours. The genes up-regulated in both MCF-7 and MDA-MB-231 cell lines, either in 6.0 μM and 12.0 μM CTet treatment conditions, were selected using GeneSifter software. The software analysis allowed to identify a list of 116 genes significantly (p < 0.01) up-regulated in both cell lines. [file bcr2855-S3.PDF]

Table 1S. List of 116 genes commonly up-regulated in treated MCF-7 and MDA-MB-231 cell lines (search pattern: control =1; 6 μM >1; 12 μM >2) (*p*<0.01)

Gene Identifier	Gene Title	Gene ID
NM_000584	interleukin 8 (IL8)	IL8
NM_005564	lipocalin 2 (oncogene 24p3) (LCN2)	LCN2
NM_004024	activating transcription factor 3 (ATF3)	ATF3
NM_000600	interleukin 6 (interferon, beta 2) (IL6)	IL6
BQ430788	AGENCOURT_7776027 NIH_MGC_68 cDNA clone IMAGE:6024295 5'	-
NM_000963	prostaglandin-endoperoxide synthase 2 (prostaglandin G/H synthase and cyclooxygenase) (PTGS2)	PTGS2
NM_001353	aldo-keto reductase family 1, member C1 (dihydrodiol dehydrogenase 1; 20-alpha (3-alpha)-hydroxysteroid dehydrogenase) (AKR1C1)	AKR1C1
NM_002133	heme oxygenase (decycling) 1 (HMOX1)	HMOX1
NM_019058	DNA-damage-inducible transcript 4 (DDIT4)	DDIT4
NM_003733	2'-5'-oligoadenylate synthetase-like (OASL), transcript variant 1	OASL
NM_001549	interferon-induced protein with tetratricopeptide repeats 4 (IFIT4)	IFIT3
NM_018295	hypothetical protein FLJ11000 (FLJ11000)	TMEM140
NM_003764	syntaxin 11 (STX11)	STX11
NM_024111	hypothetical protein MGC4504 (MGC4504)	CHAC1
NM_002201	interferon stimulated gene 20kDa (ISG20)	ISG20
NM_001548	interferon-induced protein with tetratricopeptide repeats 1 (IFIT1), mRNA	IFIT1
S62138	TLS/CHOP=hybrid gene {translocation breakpoint} [human, myxoid liposarcomas cells, mRNA Mutant, 1682 nt]	FUS
NM_005345	heat shock 70kDa protein 1A (HSPA1A)	HSPA1A
NM_006417	interferon-induced protein 44 (IFI44)	IFI44
NM_014417	BCL2 binding component 3 (BBC3)	BBC3
NM_012328	DnaJ (Hsp40) homolog, subfamily B, member 9 (DNAJB9)	DNAJB9
NM_024625	zinc finger CCCH type, antiviral 1 (ZC3HAV1), transcript variant 2	ZC3HAV1
NM_031412	GABA(A) receptor-associated protein like 1 (GABARAPL1)	GABARAPL1
NM_182743	thioredoxin reductase 1 (TXNRD1), transcript variant 4	TXNRD1
NM_014470	Rho family GTPase 1 (RND1)	RND1
NM_203418	Down syndrome critical region gene 1 (DSCR1), transcript variant 3	RCAN1
NM_197955	normal mucosa of esophagus specific 1 (NMES1), transcript variant 1	C15orf48
BC063507	heat shock 70kDa protein 1B, mRNA (cDNA clone MGC:74463 IMAGE:4153196)	HSPA1A
NM_005346	heat shock 70kDa protein 1B (HSPA1B)	HSPA1A
NM_001547	interferon-induced protein with tetratricopeptide repeats 2 (IFIT2)	IFIT2

NM_019891	ERO1-like beta (S cerevisiae) (ERO1LB)	ERO1LB
AW193472	xl75f11x1 NCI_CGAP_Pan1 cDNA clone IMAGE:2680557 3' similar to SW:IMA2_XENLA P52171 IMPORTIN ALPHA-2 SUBUNIT ;	-
CB163556	K-EST0224449 L17N670205n1 cDNA clone L17N670205n1-32-F09 5'	SFRS1
NM_004221	natural killer cell transcript 4 (NK4)	IL32
NM_031419	molecule possessing ankyrin repeats induced by lipopolysaccharide (MAIL), homolog of mouse (MAIL)	NFKBIZ
NM_005195	CCAAT/enhancer binding protein (C/EBP), delta (CEBPD)	CEBPD
NM_007034	DnaJ (Hsp40) homolog, subfamily B, member 4 (DNAJB4)	DNAJB4
NM_005194	CCAAT/enhancer binding protein (C/EBP), beta (CEBPB)	CEBPB
NM_025079	hypothetical protein FLJ23231 (FLJ23231)	ZC3H12A
NM_000104	cytochrome P450, family 1, subfamily B, polypeptide 1 (CYP1B1)	CYP1B1
NM_017983	hypothetical protein FLJ10055 (FLJ10055)	WIPI1
NM_003806	harakiri, BCL2 interacting protein (contains only BH3 domain) (HRK)	HRK
NM_006813	proline-rich nuclear receptor coactivator 1 (PNRC1)	PNRC1
AK096014	cDNA FLJ38695 fis, clone KIDNE2001897	LOC100131067
NM_007207	dual specificity phosphatase 10 (DUSP10), transcript variant 1	DUSP10
NM_130469	jun dimerization protein 2 (JDP2)	JDP2
AF001893	MEN1 region clone epsilon/beta mRNA, 3' fragment	NCRNA00084
NM_000636	superoxide dismutase 2, mitochondrial (SOD2)	SOD2
NM_005980	S100 calcium binding protein P (S100P)	S100P
NM_002985	chemokine (C-C motif) ligand 5 (CCL5)	CCL5
NM_203411	similar to RIKEN cDNA 2600017H02 (LOC92162)	TMEM88
NM_182775	hypothetical protein LOC259173 (FLJ36525), transcript variant 3	ALS2CL
NM_133436	asparagine synthetase (ASNS), transcript variant 1	ASNS
AK022035	cDNA FLJ11973 fis, clone HEMBB1001221	-
NM_006145	DnaJ (Hsp40) homolog, subfamily B, member 1 (DNAJB1)	DNAJB1
NM_001621	aryl hydrocarbon receptor (AHR)	AHR
NM_144593	Ras homolog enriched in brain like 1 (RHEBL1)	RHEBL1
BU185583	AGENCOURT_7975395 NIH_MGC_110 cDNA clone IMAGE:6082482 5'	TRIM16L
BU942678	AGENCOURT_10555965 NIH_MGC_127 cDNA clone IMAGE:6715786 5'	SQSTM1
NM_021158	tribbles homolog 3 (Drosophila) (TRIB3)	TRIB3
NM_000389	cyclin-dependent kinase inhibitor 1A (p21, Cip1) (CDKN1A), transcript variant 1	CDKN1A
NM_182491	hypothetical protein LOC90637 (LOC90637)	ZFAND2A
AK026808	cDNA: FLJ23155 fis, clone LNG09573	HERC4
NM_006096	N-myc downstream regulated gene 1 (NDRG1)	NDRG1
AK093770	cDNA FLJ36451 fis, clone THYMU2013757	9-Sep
NM_015271	tripartite motif-containing 2 (TRIM2)	TRIM2
NM_001710	B-factor, properdin (BF)	CFB
BX104647	Soares_NhHMPu_S1 cDNA clone IMAGp998A034742	-
BX648200	mRNA; cDNA DKFZp779C0742 (from clone DKFZp779C0742)	-

NM_004235	Kruppel-like factor 4 (gut) (KLF4)	KLF4
NM_005658	TNF receptor-associated factor 1 (TRAF1)	TRAF1
BX647655	mRNA; cDNA DKFZp451A211 (from clone DKFZp451A211)	DKFZp451A211
NM_177478	mitochondrial ferritin (MTF)	FTMT
AK023614	cDNA FLJ13552 fis, clone PLACE1007218	LOC100128988
AK091927	cDNA FLJ34608 fis, clone KIDNE2013845	SFRS3
NM_007076	Huntingtin interacting protein E (HYPE)	FICD
NM_012257	HMG-box transcription factor 1 (HBP1)	HBP1
CD049690	AGENCOURT_13973532 NIH_MGC_172 Homo sapiens cDNA 5'	-
NM_021127	phorbol-12-myristate-13-acetate-induced protein 1 (PMAIP1)	PMAIP1
NM_030754	serum amyloid A2 (SAA2)	SAA2
NM_002970	spermidine/spermine N1-acetyltransferase (SAT)	SAT1
AA854620	aj76f05s1 Soares_parathyroid_tumor_NbHPA cDNA clone IMAGE:1402401 3' similar to contains Alu repetitive element;	KLHDC7B
NM_005194	CCAAT/enhancer binding protein (C/EBP), beta (CEBPB)	CEBPB
NM_018602	DnaJ (Hsp40) homolog, subfamily A, member 4 (DNAJA4)	DNAJA4
N47412	yy88d12s1 Soares_multiple_sclerosis_2NbHMSP cDNA clone IMAGE:280631 3' similar to gb:L20941 FERRITIN HEAVY CHAIN (HUMAN);	FTH1
NM_020347	leucine zipper transcription factor-like 1 (LZTFL1)	LZTFL1
NM_004235	Kruppel-like factor 4 (gut) (KLF4)	KLF4
NM_006058	TNFAIP3 interacting protein 1 (TNIP1)	TNIP1
BX119436	NCI_CGAP_Brn23 cDNA clone IMAGp998G215173 ; IMAGE:2098220	CECR2
BC064405	membrane associated transporter, mRNA (cDNA clone MGC:74426 IMAGE:5563039)	SLC45A2
N34332	yy52h12s1 Soares_multiple_sclerosis_2NbHMSP cDNA clone IMAGE:277223 3'	RPS6KA2
NM_133328	death effector domain containing 2 (DEDD2)	DEDD2
NM_032621	brain expressed X-linked 2 (BEX2)	BEX2
NM_000815	gamma-aminobutyric acid (GABA) A receptor, delta (GABRD)	GABRD
BC023640	clone IMAGE:4890344	-
NM_022168	melanoma differentiation associated protein-5 (MDA5)	IFIH1
NM_030594	cytoplasmic polyadenylation element binding protein 1 (CPEB1)	CPEB1
BQ890702	AGENCOURT_8673352 Lupski_sciatic_nerve cDNA clone IMAGE:6199646 5'	-
NM_019025	spermine oxidase (SMOX), transcript variant 5	-
NM_024970	hypothetical protein FLJ11722 (FLJ11722), mRNA	-
NM_004563	phosphoenolpyruvate carboxykinase 2 (mitochondrial) (PCK2)	PCK2
NM_003364	uridine phosphorylase 1 (UPP1), transcript variant 1	UPP1
AK022339	cDNA FLJ12277 fis, clone MAMMA1001711	-
AV741130	CB cDNA clone CBCATB06 5'	-
NM_172230	HRD1 protein (HRD1), transcript variant 2	SYVN1
BG530123	602558609F1 NIH_MGC_61 cDNA clone IMAGE:4696586 5'	-
AW851082	IL3-CT0220-150200-070-A05 CT0220 Homo sapiens cDNA	-

NM_004281	BCL2-associated athanogene 3 (BAG3)	BAG3
NM_182936	solute carrier family 8 (sodium-calcium exchanger), member 3 (SLC8A3), transcript variant f	SLC8A3
BU626416	UI-H-FT0-bhm-c-16-0-UIs1 NCI_CGAP_FT0 cDNA clone UI-H-FT0-bhm-c-16-0-UI 3'	RPLP2
NM_000930	plasminogen activator, tissue (PLAT), transcript variant 1	PLAT
NM_006169	nicotinamide N-methyltransferase (NNMT)	NNMT
NM_022818	microtubule-associated protein 1 light chain 3 beta (MAP1LC3B)	MAP1LC3B
NM_012323	v-maf musculoaponeurotic fibrosarcoma oncogene homolog F (avian) (MAFF), transcript variant 1	MAFF
NM_002395	malic enzyme 1, NADP(+)-dependent, cytosolic (ME1)	ME1
NM_021101	claudin 1 (CLDN1)	CLDN1
