# Supplementary material for: The indole-3-carbinol cyclic tetrameric derivative CTet inhibits cell proliferation via overexpression of p21/CDKN1A in both estrogen receptor-positive and triple-negative breast cancer cell lines
Source: Breast Cancer Res. 2011 Mar 24;13(2):R33. doi: 10.1186/bcr2855 (PMC3219196; doi:10.1186/bcr2855)
Supplement: Additional file 4 — Table S2. Transcriptome analysis was performed on MCF-7 and MDA-MB-231 cells treated with 6.0 μM and 12.0 μM CTet for 24 hours. The genes down-regulated in both MCF-7 and MDA-MB-231 cell lines, either in 6.0 μM and 12.0 μM CTet treatment conditions, were selected using GeneSifter software. The software analysis allowed to identify a list of 177 genes significantly (p < 0.01) down-regulated in both cell lines. [file bcr2855-S4.PDF]

Table 2S. List of 177 genes commonly down-regulated in treated MCF-7 and MDA cell lines (search pattern: control =1; 6 μM <1; 12 μM <0.5) (*p*<0.01)

Gene Identifier	Gene Title	Gene ID
XM_060563	similar to seven transmembrane helix receptor (LOC127608), mRNA	-
NM_014428	tight junction protein 3 (zona occludens 3) (TJP3)	TJP3
NM_001874	carboxypeptidase M (CPM), transcript variant 1	CPM
NM_006044	histone deacetylase 6 (HDAC6)	HDAC6
NM_032297	hypothetical protein DKFZp761D112 (DKFZp761D112), mRNA	-
NM_016357	epithelial protein lost in neoplasm beta (EPLIN)	LIMA1
NM_031461	CocoaCrisp (LOC83690)	CRISPLD1
NM_003500	acyl-Coenzyme A oxidase 2, branched chain (ACOX2)	ACOX2
NM_003546	histone 1, H4l (HIST1H4L)	HIST1H4L
AK025839	cDNA: FLJ22186 fis, clone HRC01010	TMEM19
NM_001262	cyclin-dependent kinase inhibitor 2C (p18, inhibits CDK4) (CDKN2C), transcript variant 1	CDKN2C
NM_001552	insulin-like growth factor binding protein 4 (IGFBP4)	IGFBP4
NM_006479	RAD51-interacting protein (PIR51)	RAD51AP1
BG121512	602352845F1 NIH_MGC_90 cDNA clone IMAGE:4451051 5'	-
NM_024680	FLJ23311 protein (FLJ23311)	E2F8
NM_003068	snail homolog 2 (Drosophila) (SNAI2)	SNAI2
NM_020675	kinetochore protein Spc25 (Spc25)	SPC25
AK021443	cDNA FLJ11381 fis, clone HEMBA1000501	-
AK025766	cDNA: FLJ22113 fis, clone HEP18418	BRI3BP
NM_016095	DNA replication complex GINS protein PSF2 (Pfs2)	GINS2
AF111801	MSTP020 (MST020) mRNA	TRERF1
AK096284	cDNA FLJ38965 fis, clone NT2RI2000987, highly similar to Lunatic fringe precursor	LFNG
NM_002388	MCM3 minichromosome maintenance deficient 3 (S cerevisiae) (MCM3)	MCM3
AI808321	wf54d12x1 Soares_NFL_T_GBC_S1 cDNA clone IMAGE:2359415 3'	-
NM_024838	threonine synthase-like 1 (bacterial) (THNSL1)	THNSL1
NM_005573	lamin B1 (LMNB1)	LMNB1
NM_024908	hypothetical protein FLJ12973 (FLJ12973)	WDR76
NM_018518	MCM10 minichromosome maintenance deficient 10 (S cerevisiae) (MCM10), transcript variant 2	MCM10
NM_005855	receptor (calcitonin) activity modifying protein 1 (RAMP1)	RAMP1
NM_014109	ATPase family, AAA domain containing 2 (ATAD2)	ATAD2
BG286743	602381785F1 NIH_MGC_93 cDNA clone IMAGE:4499421 5'	GPATCH4
NM_016619	placenta-specific 8 (PLAC8)	PLAC8

NM_024094	defective in sister chromatid cohesion homolog 1 (S cerevisiae) (MGC5528)	DSCC1
NM_004702	cyclin E2 (CCNE2), transcript variant 3	-
NM_024955	hypothetical protein FLJ23322 (FLJ23322)	FOXRED2
NM_018485	G protein-coupled receptor 77 (GPR77)	GPR77
NM_001798	cyclin-dependent kinase 2 (CDK2), transcript variant 1	CDK2
NM_002961	S100 calcium binding protein A4 (calcium protein, calvasculin, metastasin, murine placental homolog) (S100A4), transcript variant 1	S100A4
NM_004091	E2F transcription factor 2 (E2F2)	E2F2
BX538051	mRNA; cDNA DKFZp686F09156 (from clone DKFZp686F09156)	LOC730102
NM_001071	thymidylate synthetase (TYMS)	TYMS
NM_001948	dUTP pyrophosphatase (DUT)	DUT
NM_173554	chromosome 10 open reading frame 107 (C10orf107)	C10orf107
AK126467	cDNA FLJ44503 fis, clone UTERU3001158	-
AI198829	qf54f03x1 Soares_testis_NHT cDNA clone IMAGE:1753853 3'	-
AF131841	clone 24974 mRNA sequence	-
AW102825	xd38g04x1 NCI_CGAP_Ov23 cDNA clone IMAGE:2596086 3'	-
N28812	yx71b12r1 Soares melanocyte 2NbHM cDNA clone IMAGE:267167 5'	CYP2U1
BU429796	UI-HF-BN0-aff-g-01-0-UIr1 NIH_MGC_50 cDNA clone IMAGE:3066792 5'	NBEAL2
NM_145307	pleckstrin homology domain containing, family K member 1 (PLEKHK1)	RTKN2
BX648959	mRNA; cDNA DKFZp686N2348 (from clone DKFZp686N2348)	C1orf21
NM_016463	CXXC finger 5 (CXXC5)	CXXC5
BQ010979	UI-1-BC1p-asr-c-12-0-UIs1 NCI_CGAP_Pl3 cDNA clone UI-1-BC1p-asr-c-12-0-UI 3'	-
NM_018279	transmembrane protein 19 (TMEM19)	TMEM19
AK057253	cDNA FLJ32691 fis, clone TESTI2000221	-
H97329	EST48b105 WATM1 cDNA clone 48b105	-
BE788763	601475864F1 NIH_MGC_68 cDNA clone IMAGE:3879014 5'	-
NM_138970	neurexin 3 (NRXN3), transcript variant beta	NRXN3
NM_207380	FLJ43339 protein (FLJ43339)	C15orf52
AI738835	tr28g10x1 NCI_CGAP_Ov23 cDNA clone IMAGE:2219682 3' similar to contains element MER2 repetitive element ;	-
R89137	yp99e07r1 Soares fetal liver spleen 1NFLS cDNA clone IMAGE:195588 5'	EIF4A2
NM_016310	polymerase (RNA) III (DNA directed) polypeptide K, 123 kDa (POLR3K)	POLR3K
NM_002775	protease, serine, 11 (IGF binding) (PRSS11)	HTRA1
AK096284	cDNA FLJ38965 fis, clone NT2RI2000987, highly similar to Lunatic fringe precursor	LFNG
CN256561	17000599944328 GRN_PREHEP Homo sapiens cDNA 5'	-
NM_018894	EGF-containing fibulin-like extracellular matrix protein 1 (EFEMP1), transcript variant 2	-
NM_025004	hypothetical protein FLJ13215 (FLJ13215)	CCDC15

BF207862	601862570F1 NIH_MGC_53 cDNA clone IMAGE:4082080 5'	-
NM_181578	replication factor C (activator 1) 5, 365kDa (RFC5), transcript variant 2	RFC5
BF508354	UI-H-BI4-aqb-e-06-0-UI.s1 NCI_CGAP_Sub8 cDNA clone IMAGE:3089363 3'	C21orf58
NM_006392	nucleolar protein 5A (56kDa with KKE/D repeat) (NOL5A)	NOP56
NM_006461	sperm associated antigen 5 (SPAG5)	SPAG5
NM_015035	zinc fingers and homeoboxes 3 (ZHX3)	ZHX3
NM_032510	par-6 partitioning defective 6 homolog gamma (C elegans) (PARD6G)	PARD6G
AK024851	cDNA: FLJ21198 fis, clone COL00220	-
NM_014065	HT001 protein (HT001)	ASTE1
NM_012137	dimethylarginine dimethylaminohydrolase 1 (DDAH1)	DDAH1
NM_001333	cathepsin L2 (CTSL2)	CTSL2
AK128700	cDNA FLJ46867 fis, clone UTERU3012293, weakly similar to Homo sapiens zinc finger protein 14 (KOX 6) (ZNF14)	ZNF788
NM_001439	exostoses (multiple)-like 2 (EXTL2)	EXTL2
BU624025	UI-H-FG1-bgh-j-10-0-UIs1 NCI_CGAP_FG1 cDNA clone UI-H-FG1-bgh-j-10-0-UI 3'	IL31RA
NM_144781	programmed cell death 2 (PDCD2), transcript variant 2	PDCD2
NM_002692	polymerase (DNA directed), epsilon 2 (p59 subunit) (POLE2)	POLE2
BX648964	mRNA; cDNA DKFZp686J0156 (from clone DKFZp686J0156)	GFRA1
BX647730	mRNA; cDNA DKFZp686O0225 (from clone DKFZp686O0225)	ZADH2
NM_003539	histone 1, H4d (HIST1H4D)	HIST1H4D
AA947258	od86c08s1 NCI_CGAP_Ov2 cDNA clone IMAGE:1374830	-
NM_025268	hole gene (MGC4659)	TMEM121
NM_001878	cellular retinoic acid binding protein 2 (CRABP2)	CRABP2
BC033086	transcription factor 19 (SC1), mRNA (cDNA clone MGC:45652 IMAGE:3160434)	TCF19
BX647688	mRNA; cDNA DKFZp779C093 (from clone DKFZp779C093)	SYTL5
D29134	HUMNK158 Human epidermal keratinocyte cDNA clone 158	-
NM_016048	CGI-111 protein (CGI-111)	ISOC1
NM_006276	splicing factor, arginine/serine-rich 7, 35kDa (SFRS7)	-
AK128460	cDNA FLJ46603 fis, clone THYMU3047513	-
NM_005450	noggin (NOG)	NOG
NM_133334	Wolf-Hirschhorn syndrome candidate 1 (WHSC1), transcript variant 7	WHSC1
NM_015493	ankyrin repeat domain 25 (ANKRD25)	KANK2
NM_000998	ribosomal protein L37a (RPL37A)	RPL37A
NM_000692	aldehyde dehydrogenase 1 family, member B1 (ALDH1B1), nuclear gene encoding mitochondrial protein	ALDH1B1
NM_007057	ZW10 interactor (ZWINT), transcript variant 1	ZWINT
NM_183065	hypothetical protein MGC10744 (MGC10744), transcript variant 2	TMEM107
U00930	clone C4E 163 (CAC)n/(GTG)n repeat-containing mRNA.	CHD8

BM670793	UI-E-DX1-agv-p-03-0-UIs1 UI-E-DX1 cDNA clone UI-E-DX1-agv-p-03-0-UI 3'	TTC12
AB046806	KIAA1586 protein	-
AL833129	mRNA; cDNA DKFZp313I1032 (from clone DKFZp313I1032)	-
AA527854	nh85g09s1 NCI_CGAP_Br1.1 cDNA clone IMAGE:965344 3'	-
BC014312	histone 1, H2bj, mRNA (cDNA clone MGC:22655 IMAGE:4048288)	-
NM_012415	RAD54 homolog B (S cerevisiae) (RAD54B), transcript variant 1	RAD54B
NM_015631	chromosome 10 open reading frame 61 (C10orf61)	TCTN3
NM_016441	cysteine-rich motor neuron 1 (CRIM1)	CRIM1
NM_032285	hypothetical protein MGC3207 (MGC3207)	MRI1
NM_152540	sec1 family domain containing 2 (SCFD2)	SCFD2
NM_024532	PF20 (PF20)	SPAG16
NM_022770	hypothetical protein FLJ13912 (FLJ13912)	GINS3
NM_033502	transcriptional regulating factor 1 (TRERF1), transcript variant 1	TRERF1
NM_005463	heterogeneous nuclear ribonucleoprotein D-like (HNRPDL), transcript variant 1	-
NM_000216	Kallmann syndrome 1 sequence (KAL1)	KAL1
NM_004203	membrane-associated tyrosine- and threonine-specific cdc2-inhibitory kinase (PKMYT1), transcript variant 1	PKMYT1
NM_153267	MAM domain containing 2 (MAMDC2)	MAMDC2
NM_006793	peroxiredoxin 3 (PRDX3), nuclear gene encoding mitochondrial protein, transcript variant 1	PRDX3
NM_007243	nurim (nuclear envelope membrane protein) (NRM)	NRM
BE900490	601673740F1 NIH_MGC_21 cDNA clone IMAGE:3956600 5'	DGKZ
AB007969	mRNA, chromosome 1 specific transcript KIAA0500	CLMN
NM_002250	potassium intermediate/small conductance calcium-activated channel, subfamily N, member 4 (KCNN4)	KCNN4
BM993871	UI-H-DF1-aug-h-10-0-UIs1 NCI_CGAP_DF1 cDNA clone IMAGE:5869113 3'	HDAC2
NM_024503	immunodeficiency virus type I enhancer binding protein 3 (HIVEP3)	HIVEP3
AB007976	mRNA, chromosome 1 specific transcript KIAA0507	RRP15
NM_000310	palmitoyl-protein thioesterase 1 (ceroid-lipofuscinosis, neuronal 1, infantile) (PPT1)	PPT1
AK023647	cDNA FLJ13585 fis, clone PLACE1009150	-
BX090418	Soares fetal liver spleen 1NFLS cDNA clone IMAGp998J03131 ; IMAGE:127970	-
NM_138484	shugoshin-like 1 (S pombe) (SGOL1)	SGOL1
NM_005148	unc-119 homolog (C elegans) (UNC119), transcript variant 1	UNC119
NM_024330	solute carrier family 27 (fatty acid transporter), member 3 (SLC27A3)	SLC27A3
NM_030937	cyclin L2 (CCNL2)	CCNL2
NM_145006	sushi domain containing 3 (SUSD3)	SUSD3

NM_024534	hypothetical protein FLJ12684 (FLJ12684)	-
AK092442	cDNA FLJ35123 fis, clone PLACE6008036	PP14571
NM_000186	H factor 1 (complement) (HF1)	CFH
BC043001	clone IMAGE:5297432	LOC401176
NM_058187	chromosome 21 open reading frame 63 (C21orf63)	C21orf63
NM_138443	coiled-coil domain containing 5 (spindle associated) (CCDC5)	HAUS1
NM_022337	RAB38, member RAS oncogene family (RAB38)	RAB38
D28589	KIAA00167 mRNA, partial sequence	KIAA0114
NM_030763	nucleosomal binding protein 1 (NSBP1)	NSBP1
AB037863	KIAA1442 protein	-
NM_198327	suppression of tumorigenicity 7 like (ST7L), transcript variant 5	-
BM666683	UI-E-CQ1-aey-k-19-0-UIs1 UI-E-CQ1 cDNA clone UI-E-CQ1-aey-k-19-0-UI 3'	-
NM_021177	LSM2 homolog, U6 small nuclear RNA associated (S cerevisiae) (LSM2)	LSM2
-	INCYTE UNIQUE	-
NM_016551	transmembrane 7 superfamily member 3 (TM7SF3)	TM7SF3
NM_173543	hypothetical protein FLJ32844 (FLJ32844)	DZIP1L
AB033091	KIAA1265 protein	SLC39A10
NM_020226	PR domain containing 8 (PRDM8)	PRDM8
BM715449	UI-E-EJ0-ahi-e-22-0-UIr2 UI-E-EJ0 cDNA clone UI-E-EJ0-ahi-e-22-0-UI 5'	ZNF618
R67468	yi33b11r1 Soares placenta Nb2HP cDNA clone IMAGE:141021 5'	-
NM_194276	hypothetical protein FLJ20209 (FLJ20209)	-
NM_004242	high mobility group nucleosomal binding domain 3 (HMGN3), transcript variant 1	HMGN3
NM_004442	EphB2 (EPHB2), transcript variant 2	EPHB2
NM_016448	RA-regulated nuclear matrix-associated protein (RAMP)	DTL
NM_001002295	GATA binding protein 3 (GATA3), transcript variant 1, mRNA	GATA3
NM_005424	tyrosine kinase with immunoglobulin and epidermal growth factor homology domains (TIE)	TIE1
NM_198920	chromosome 6 open reading frame 157 (C6orf157)	UBE2CBP
NM_006198	Purkinje cell protein 4 (PCP4)	PCP4
AA844712	ai70e12s1 Soares_testis_NHT cDNA clone IMAGE:1376206 3'	FAT3
NM_005915	MCM6 minichromosome maintenance deficient 6 (MIS5 homolog, S pombe) (S. cerevisiae) (MCM6)	MCM6
N98647	yy66a04r1 Soares_multiple_sclerosis_2NbHMSP cDNA clone IMAGE:278478 5'	-
NM_015577	retinoic acid induced 14 (RAI14)	RAI14
AA150617	zl43h08r1 Soares_pregnant_uterus_NbHPU cDNA clone IMAGE:504735 5'	LYPD6
CA417327	UI-H-FE0-bbt-h-18-0-UI.s1 NCI_CGAP_FE0 cDNA clone UI-H-FE0-bbt-h-18-0-UI 3'	-
R98802	yr32a08s1 Soares fetal liver spleen 1NFLS cDNA clone IMAGE:206966 3'	ACACB

NM_005012	receptor tyrosine kinase-like orphan receptor 1 (ROR1)	ROR1
NM_006411	1-acylglycerol-3-phosphate O-acyltransferase 1 (lysophosphatidic acid acyltransferase, alpha) (AGPAT1), transcript variant 1	AGPAT1
AK026323	cDNA: FLJ22670 fis, clone HSI08684	ALPK1
NM_001159	aldehyde oxidase 1 (AOX1)	AOX1
NM_003022	SH3 domain binding glutamic acid-rich protein like (SH3BGRL)	SH3BGRL
NM_024573	chromosome 6 open reading frame 211 (C6orf211)	C6orf211
