# Supplementary material for: The indole-3-carbinol cyclic tetrameric derivative CTet inhibits cell proliferation via overexpression of p21/CDKN1A in both estrogen receptor-positive and triple-negative breast cancer cell lines
Source: Breast Cancer Res. 2011 Mar 24;13(2):R33. doi: 10.1186/bcr2855 (PMC3219196; doi:10.1186/bcr2855)
Supplement: Additional file 6 — Table S3. Antiproliferative activity of CTet in different storing conditions in MCF-7 cells. Aliquots of CTet were stored at different conditions of temperature and light exposition and the activity was then evaluated in MCF-7 at different time points (4, 8, 12, 20 weeks and after one year). Results are reported as IC50 values. [file bcr2855-S6.PDF]

Table 3S.

Antiproliferative activity in MCF-7 cells of CTet in different storing conditions

	Vehicle	Temperature	Light Exposure	IC_{50} (µM) [a]				
				4 week	8 week	12 week	20 week	1 year
1.	Pure EtOH [b]	RT [c]	Light	2.7	1.9	1.9	2.9	2.4
2.	Pure EtOH	RT	Dark	2.3	2.1	1.6	2.9	1.5
3.	γ-CD (10% EtOH)	RT	Light	1.2	1.0	1.3	1.6	0.9
4.	γ-CD (10% EtOH)	RT	Dark	1.1	0.8	0.8	1.2	0.7
5.	Pure EtOH	+ 4°C	Dark	0.9	0.9	1.1	1.4	1.2
6.	γ-CD (10% EtOH)	+ 4°C	Dark	1.1	0.9	0.7	1.1	0.8

[a] IC_{50} at time 0 in MCF-7 cells: 1.4 µM CTet.

[b] CTet stored in pure Ethanol were diluted 1:10 in γ-cyclodextrin solution immediately before the antiproliferative assays.

[c] RT: Room temperature.
